# Methylation of *SDC2*/*TFPI2* and Its Diagnostic Value in Colorectal Tumorous Lesions

**DOI:** 10.3389/fmolb.2021.706754

**Published:** 2021-12-22

**Authors:** Lianglu Zhang, Lanlan Dong, Changming Lu, Wenxian Huang, Cuiping Yang, Qian Wang, Qian Wang, Ruixue Lei, Rui Sun, Kangkang Wan, Tingting Li, Fan Sun, Tian Gan, Jun Lin, Lei Yin

**Affiliations:** ^1^ Department of Biochemistry, College of Life Sciences, Wuhan University, Wuhan, China; ^2^ Wuhan Ammunition Life-tech Company, Ltd., Wuhan, China; ^3^ Department of Gastroenterology, Zhongnan Hospital of Wuhan University, Wuhan, China; ^4^ The Hubei Clinical Center and Key Laboratory of Intestinal and Colorectal Diseases, Wuhan, China; ^5^ Department of Pathology, Renmin Hospital of Wuhan University, Wuhan, China; ^6^ Department of Gastroenterology, Ruijin Hospital North, Shanghai Jiaotong University School of Medicine, Shanghai, China; ^7^ Department of Colorectal and Anal Surgery, The Eighth Hospital of Wuhan, Hubei University of Chinese Medicine, Wuhan, China; ^8^ Department of Pathology, The Fourth Affiliated Hospital of Henan University of Science and Technology (Anyang Tumor Hospital), Anyang, China; ^9^ Department of Oncology, Wuhan Fourth Hospital (Puai Hospital), Tongji Medical College, Huazhong University of Science and Technology, Wuhan, China

**Keywords:** colorectal cancer, adenomas, SDC2, TFPI2, methylation

## Abstract

**Background:** *SDC2* methylation is a feasible biomarker for colorectal cancer detection. Its specificity for colorectal cancer is higher than 90%, but the sensitivity is normally lower than 90%. This study aims to improve the sensitivity of *SDC2* detection through finding a high positive target from the false-negative samples of *SDC2* detection based on analysis of the bowel subsite difference in methylation.

**Methods:** Hypermethylated *TFPI2* was identified in *SDC2* hypomethylated colorectal cancer samples retrieved from TCGA database with the methylation level lower than 0.2. The methylation-specific PCR assay was developed and then evaluated using tissue samples (184 cancer and 54 healthy control samples) and stool samples (289 cancer, 190 adenoma, and 217 healthy control samples).

**Results:** *TFPI2* was hypermethylated in most *SDC2* hypomethylated colorectal cancer samples. When the *SDC2*/*TFPI2*-combined PCR assay was performed in stool specimens, the AUC value of cancer vs. control was 0.98, with the specificity of 96.40% and sensitivity of 96.60%, and the AUC value of adenoma vs. control was 0.87, with the specificity of 95.70% and the sensitivity of 80.00%. The improvement in sensitivity was the most momentous in the left colon. As the detection index, the Ct value was better in improving the sensitivity of detection than the methylation level based on the 2^−ΔΔCt^ value.

**Conclusion:** *TFPI2* can improve the sensitivity of *SDC2* methylation–specific detection of colorectal tumorous lesions while maintaining high specificity, in particular reducing the missed detection of left colon cancer and adenoma.

## Introduction

Colorectal cancer (CRC) affects millions of people around the world. It is unique because of slow progress, making it preventable and often curable (Cao et al., 2021; Sung et al., 2021). The five-year survival rate can be as high as 90% or less than 10%, depending on the stage of diagnosis (American Cancer Society, 2019). Sporadic CRC mainly develops in a normal–adenoma–carcinoma sequence (Crockett and Nagtegaal, 2019), and early detection can significantly decrease mortality (Chung, 2018). Colonoscopy plus pathological examination is the gold standard for CRC diagnosis (Shaukat et al., 2021), but due to the invasive and complex intestinal preparation process, its compliance in the average risk population is low (Navarro et al., 2017). The fecal occult blood test (FOBT) and fecal immunochemical test (FIT) are non-invasive, but their sensitivity is insufficient, especially for stage I CRC and advanced adenomas (Werner et al., 2016). The occurrence of CRC is related to genomic and epigenetic changes, such as gene mutation, microsatellite instability, and CpG island aberrant methylation (Grady and Pritchard, 2014). Among them, CpG island methylation is the most common change (Yiu and Yiu, 2016).

Aberrant DNA methylation can occur at the very early stage (Novak et al., 2009); so far, several methylation biomarkers have been identified, including *SDC2*, *NDRG4*, *BMP3*, *VIM*, *SFRP2*, and *SEPT9* (Müller et al., 2004; Loh et al., 2008; deVos et al., 2009; Melotte et al., 2009; Oh et al., 2013; Robertson and Imperiale, 2015), but the sensitivity of a single marker is usually lower than 90% (Shariatpanahi et al., 2018). The first stool-based CRC detection product “Cologuard,” targeting the hemoglobin, KRAS mutation, and two methylated genes (*NDRG4* and *BMP3*), has a sensitivity of 92% and specificity of 87% for CRC (Imperiale et al., 2014); however, multiple target tests may be costly and difficult to implement.

*SDC2* has been identified as a potential biomarker for CRC (Oh et al., 2013). Aberrant methylation in *SDC2* CpG islands has been found in tissue, blood, and stool (Oh et al., 2013; Mitchell et al., 2014). A study based on Chinese stool samples showed that the sensitivity and specificity of *SDC2* for CRC were 81.1 and 93.3%, respectively (Niu et al., 2017). Korean researchers adopted an LTE-q methylation-specific PCR (MSP) method to enrich *SDC2*, which required two rounds of PCR, i.e., unidirectional linear amplification of target DNA followed by MSP analysis of target region, giving the sensitivity of 90.0% for CRC and specificity of 90.9% (Oh et al., 2017; Han et al., 2019).

In this study, we chose an alternative approach to improve the accuracy for CRC detection. Through genome-wide screening, we found that *TFPI2* was highly hypermethylated in *SDC2* hypomethylated samples. The combined detection of *TFPI2* and *SDC2* showed both high specificity and sensitivity, especially for cancer and adenomas in the left colon for both tissue and stool specimens. Here, we present the results of *TFPI2* identification and the evaluation of MSP systems in the tissue and stool of patients with colorectal lesions at different bowel sites.

## Materials and Methods

### Patients and Sample Collection

This study was approved by the Medical Ethics Committee, Zhongnan Hospital of Wuhan University (ethical approval No. 2019099). Tissue specimens of 198 CRC patients and 54 healthy controls (Supplementary Table S1) were collected from Zhongnan Hospital of Wuhan University, and stool specimens of 289 CRC patients, 190 adenoma patients, and 217 healthy controls (Supplementary Table S2) were from Zhongnan Hospital and Renmin Hospital of Wuhan University, Ruijin Hospital of Shanghai Jiaotong University, Wuhan Eighth Hospital of Hubei University of Chinese Medicine, the Fourth Affiliated Hospital of Henan University of Science and Technology, and Wuhan Fourth Hospital of Huazhong University of Science and Technology. Written informed consent was obtained from all participants. Exclusion criteria of tissue specimens are CRC patients with a history of CRC surgery, chemotherapy, or other treatment and non-CRC patients who have received chemotherapy in the past 6 months. Finally, 184 CRC and 54 normal tissue specimens from different colorectal sites were included (Supplementary Table S1). The stool specimen exclusion criteria are patients with tumor other than CRC; patients who had a history of surgery or chemotherapy; patients with familial or hereditary colorectal adenomas or tumor; patients with non-primary tumors and other undiagnosed cases; and patients who had undergone colonic invasive surgery or bowel preparation less than 1 week before sample collection.

### Discovery of Biomarker Complementary to *SDC2*

391 CRC specimens from The Cancer Genome Atlas (TCGA) were sorted according to the methylation level (mean *β* value of the probes within the CpG island) of *SDC2* to identify hypomethylated specimens (methylation level≤0.2), and 12 selected probes (cg13096260, cg18719750, cg24732574, cg08979737, cg25070637, cg14538332, cg16935295, cg04261408, cg14625631, cg10292139, cg16673702, and cg07146119) were included. Hypermethylated genes (methylation level>0.2) were identified among hypomethylated specimens through whole-genome analysis. Receiver-operating characteristic (ROC) curve analysis was performed using the methylation level of each sample to evaluate the diagnostic complementarity of hypermethylation genes and *SDC2* in different colorectal sites.

For the samples in TCGA, if the mean *β* value of a gene was higher than 0.2, then the gene was considered to be methylation positive in this sample; otherwise, it was considered methylation negative. When two genes were analyzed jointly, as long as any gene was positive or both genes were positive at the same time, then the sample was regarded as methylation positive.

### Specimen Processing and DNA Extraction

Genomic DNA of cell lines was isolated using QIAamp DNA Mini Kit (Qiagen, Germany). All tissue specimens in this study were formalin-fixed paraffin-embedded specimens. Genomic DNA of tissue specimens was isolated using QIAamp DNA FFPE Tissue Kit (Qiagen, Germany) according to the manufacturer’s instruction.

Stool DNA was extracted using reagent developed by Wuhan Ammunition Life-tech Company. Briefly, stool specimens were collected about 8 g per person and kept in 45 ml tubes with 32 ml preservation buffer (200 mmol/L Tris·HCl, 300 mmol/L EDTA·2Na, 150 mmol/L NaCl, pH 8.0). For isolation of human genomic DNA, the biotin-labeled capture probes were designed for *SDC2*, *TFPI2*, and reference gene *ACTB*, and sequences are shown in Supplementary Table S3. After centrifugation, DNA in the supernatant was denatured under 90°C for 15 min. The single-strand DNA and the capture probes were then incubated with streptavidin magnetic beads at room temperature for 1 h. After washing twice, the target DNA was eluted with 50 uL TE buffer. All purified DNA was stored at −20°C until use.

### Bisulfite Conversion

DNA was converted using EZ DNA Methylation Kit (Zymo Research, LA, USA) according to the manufacturer’s instruction. For tissue samples, 1 ug DNA was converted; for stool samples, all the 50 ul purified DNA was converted. After bisulfite conversion, the 25 uL eluted DNA was either used immediately for PCR analysis or stored at −20°C for further use.

### Cell Lines and Plasmids

Hacat and HT-29 cell lines were used in this study; they were obtained from Tongji Medical College, Huazhong University of Science and Technology. The cell lines were cultured in DMEM (Thermo) supplemented with 10% fetal bovine serum.

The fully methylated and non-methylated amplicon regions of *SDC2* and *TFPI2* as well as the *ACTB* amplicon region after bisulfite conversion were artificially synthesized and cloned into the vector pUC57, respectively, in Wuhan GeneCreate Biological Engineering Company, and then the constructed plasmids were serially diluted to 10^6^ copies/ul, 10^5^ copies/ul, 10^4^ copies/ul, 10^3^ copies/ul, and 10^2^ copies/ul.

10^6^ copies/ul of non-methylated *SDC2* plasmids, non-methylated *TFPI2* plasmids, and *ACTB* plasmids were mixed in 1:1:1 ratio to serve as a negative control, and 10^4^ copies/uL of methylated *SDC2* plasmids, methylated *TFPI2* plasmids, and *ACTB* plasmids were mixed in 1:1:1 ratio to serve as a positive control.

### MSP

Before MSP analysis on tissue and stool samples, methylation-specific primers and probes were verified in two ways. On the one hand, 10^2^ copies/ul to 10^6^ copies/ul of methylated *SDC2* plasmids, 10^2^ copies/ul to 10^6^ copies/ul of methylated *TFPI2* plasmids, and 10^2^ copies/uL to 10^6^ copies/ul of *ACTB* plasmids were amplified to build standard curves, and amplification efficiency was calculated for each gene. On the other hand, the negative and positive controls, as well as methylated cell line (HT-29) and non-methylated cell line (Hacat), were MSP analyzed to confirm that the primers and probe could only amplify the methylated template. 500 ng genomic DNA of each cell line was added into the stool sample of healthy people confirmed by colonoscopy, and follow-up operations were the same as those of other stool samples.

Sequences of MSP primers and probes are shown in Supplementary Table S3. PCR solution was prepared in a volume of 25 ul with High-Affinity Hotstart Taq Polymerase. 5 ul template DNA was added, and non-template control and methylated and non-methylated controls were tested together in every plate. PCR was performed on an ABI 7500 instrument under the following cycling conditions: 95°C for 10 min, followed by 45 cycles of 95°C for 15 s and 60°C for 30 s.

The methylation status of *SDC2* and *TFPI2* in bisulfite-modified DNA was investigated in a blinded manner by MSP with primers specifically amplifying the methylated alleles. All MSP tests were done by investigators blinded to patients’ colonoscopy and pathology information, and MSP results were analyzed independently by other researchers.

### MSP Result Analysis

The methylation level was calculated using the formula

$$ML=2^{-\Delta \Delta Ct},$$

in which $\Delta \Delta Ct={(Ct_{t\arg et}-Ct_{ACTB)\;sample}-\left(Ct_{t\arg et}-Ct_{ACTB}\right)}_{positive\;control}$

Ct values and ML values were ROC curve analyzed separately. The optimal cutoff was determined by maximizing Youden’s index. The area under the ROC curve (AUC) value and 95% CI, sensitivity, and specificity were estimated.

When calculating the detection accuracy of marker(s) of cancer and adenoma in different colorectal sites, the cutoff value was set at Ct = 38 for *SDC2* or *TFPI2* and Ct = 36 for *ACTB*. If the Ct value of *ACTB* > 36, the reaction was invalid. The specimen was methylation positive if the Ct value of *SDC2* or/and *TFPI2* ≤ 38. Sensitivity and specificity values were calculated as

$$\begin{array}{l} \mathrm{Sensitivity}=\mathrm{methylation}\;\mathrm{positive}\;\mathrm{number}/\mathrm{total}\;\mathrm{case}\;\mathrm{number}\times 100\%,\\ \mathrm{Specificity}=\mathrm{methylation}\;\mathrm{negative}\;\mathrm{number}/\mathrm{total}\;\mathrm{control}\;\mathrm{number}\times 100\%. \end{array}$$

### Statistical Methods

All the bioinformatic and statistical analyses were performed with R version 3.6.1. To determine the statistical significance of the difference in methylation level between case and control groups, Wilcoxon’s signed-rank test was used for pairwise data and Mann–Whitney U test for groupwise data. Sensitivity between different colorectal locations was tested by the Fisher exact test.

## Results

Five groups of samples were utilized in this study, as described in Table 1. TCGA, GSE48684, and GSE79740 were used to identify and validate differential methylation regions and D184 and D289 to evaluate the performance of MSP assays in clinical samples. The TCGA dataset included 410 case and 45 control samples. We retained 391 CRC samples and 45 normal control samples for differential methylation region discovery study after removing the samples of incomplete clinical information and tumor metastasis (Supplementary Table S4). The datasets GSE48684 and GSE79740 were downloaded from the Gene Expression Omnibus (GEO) database (Luo et al., 2014; Alvi et al., 2017). GSE48684 contains 106 CRC, 42 adenoma, and 41 normal samples, and GSE79740 contains 44 CRC and 10 normal samples. They were both used for differential methylation region validation. D184 contains 184 CRC and 54 normal tissue samples, and D289 contains 289 CRC, 190 adenoma, and 217 normal stool samples. Their demographic features are shown in Supplementary Tables S1, S2.

**TABLE 1:** Description of the sample groups used in this study.

Specimen group	TCGA	GSE48684	GSE79740	D184	D289
Specimen type	Tissue	Tissue	Tissue	Tissue	Stool
Data source	GPL13534	GPL13534	GPL13534	Collected in this study	Collected in this study
Method^a^ ^,^ ^b^	450k	450k	450k	PCR	PCR
Methylation indicator	β values	β values	β values	Ct values	Ct values
Normal specimens	45	41	10	54	217
Adenoma specimens	0	42	0	0	190
CRC specimens	411	106	44	184	289
Demographic feature^c^	Table S4	NA	NA	Table S1	Table S2
Used in this study for	Marker discovery	Marker validation	Marker validation	Clinical evaluation	Clinical evaluation

a 450k means HumanMethylation450 BeadChip.

b PCR means methylation-specific PCR developed in this study.

c NA means not available here.

### High Frequency of Hypermethylated *TFPI2* Was Present in *SDC2* Hypomethylated CRC Specimens

391 CRC samples from TCGA were sorted according to the methylation level (mean *β* value of the probes) of the *SDC2* CpG island. In several previous studies on 450k methylation array, researchers elaborated the distribution characteristics of *β* and M values. Their results indicated that the bimodal distribution of M value was clearly separated when *β* value equaled 0.2. In fact, the authors directly called the peak below 0.2 the unmethylated peak and the other peak above 0.2 the methylated peak in their study, which suggested that 0.2 can be an appropriate threshold (Du et al., 2010; Dedeurwaerder et al., 2011). In this study, 50 CRC samples had a methylation level lower than 0.2 (Supplementary Table S5). Genes with a β value greater than 0.2 in these *SDC2* hypomethylated samples were retrieved throughout the whole genome, and genes complementary to *SDC2* in different colorectal sites were selected. *TFPI2* showed the best complementarities to *SDC2* in various colorectal sites (Table 2). 44 out of 50 specimens showed a *TFPI2 β* value higher than 0.2, accounting for 88.0% (Table 2).

**TABLE 2:** Detection rate of *TFPI2* in colorectal cancer tissues with the *SDC2 β* value lower than 0.2.

Tumor location	Frequency of specimens			*TFPI2* hypermethylation rate (%)
	β > 0.2	β ≤ 0.2	Total	
Splenic flexure of the colon	2	0	2	100.00
Sigmoid colon	14	1	15	93.30
Rectosigmoid junction	9	2	11	81.80
Descending colon	3	0	3	100.00
Rectum, NOS	5	0	5	100.00
Colon, NOS	4	1	5	80.00
Ascending colon	3	2	5	60.00
Hepatic flexure of the colon	1	0	1	100.00
Transverse colon	1	0	1	100.00
Connective, subcutaneous, and other soft tissues of the abdomen	1	0	1	100.00
Unknown	1	0	1	100.00
Total	44	6	50	88.00

### TFPI2 and SDC2 Were Heavily Hypermethylated in Case Rather Than Control Specimens in TCGA, GSE48684, and GSE79740 Datasets

**FIGURE 1:**
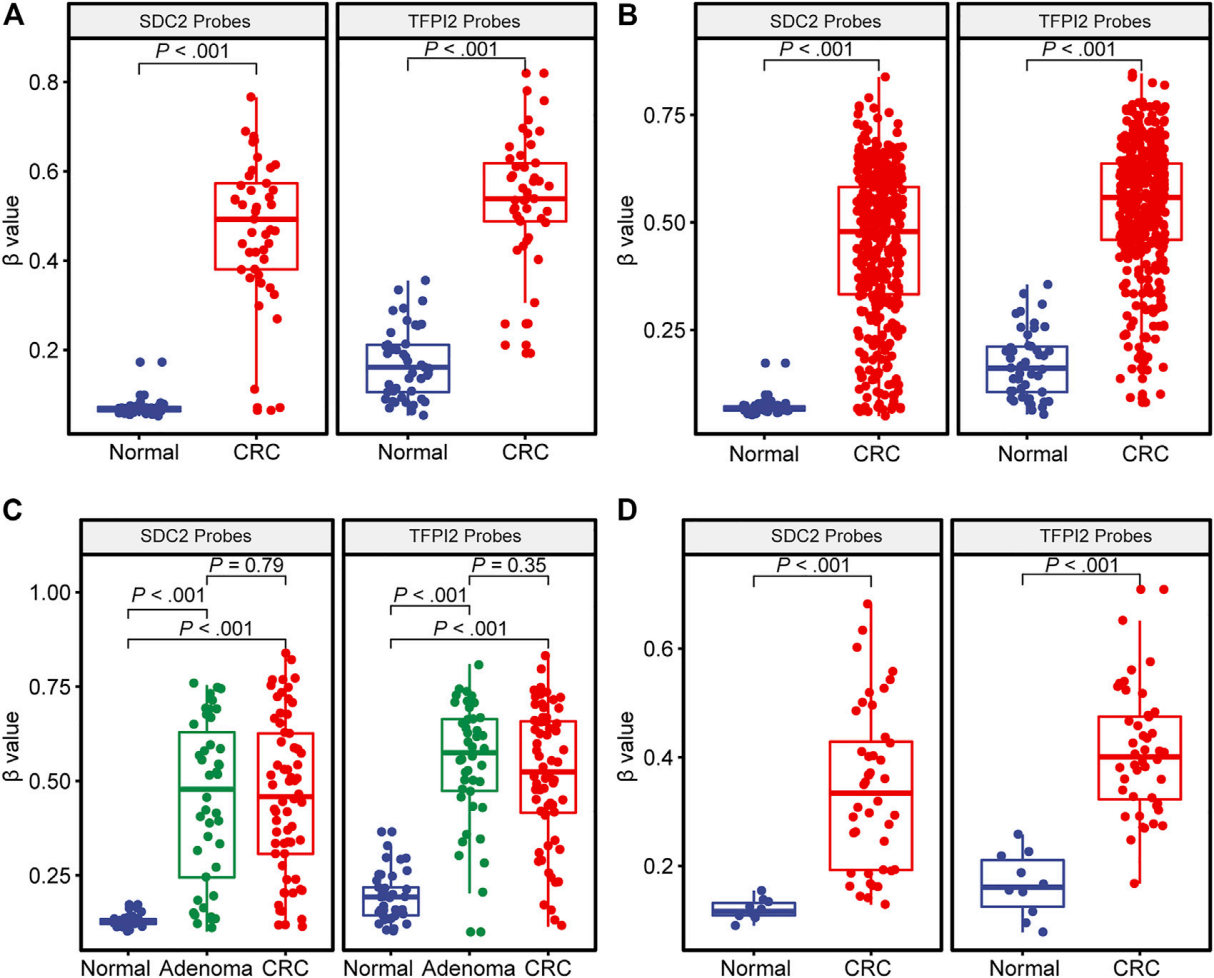
DNA methylation level (β value) of SDC2 and TFPI2 in CRC, adenoma, and normal tissues. The abscissa is the type of tissue, and the ordinate is the methylation level (β value). Each dot indicates an individual specimen. The extremes of the boxes define the upper and lower quartiles, and the center lines define the median. Whiskers indicate 1.5×interquartile range (IQR). Beyond IQR are defined the outliers. **(A)** illustrates the distribution of methylation levels in 45 CRC specimens vs. their paired normal tissue specimens from TCGA. **(B)** shows 391 CRC vs. 45 normal tissue specimens from TCGA. **(C)** is based on the dataset of GSE48684, with 106 CRC, 42 adenoma, and 41 normal tissue specimens. **(D)** is based on GSE79740, with 44 CRC and 10 normal tissue specimens. The Wilcoxon signed-rank test was used for **(A)** and Mann–Whitney U test for **(B–D)**.

The comparison of DNA methylation level (*β* value) of *SDC2* and *TFPI2* in colorectal cancer, adenoma, and normal tissues is given in Figure 1. The average methylation level of the *SDC2* gene in 45 TCGA normal tissues was 0.067, while that in 45 paired CRC tissues was 0.492 (Figure 1A). The average methylation level of the *TFPI2* gene in 45 normal tissues was 0.161, while that in paired CRC tissues was 0.538 (Figure 1A). The difference of either *SDC2* or *TFPI2* methylation level between 45 CRC tissues and adjacent normal tissues was highly significant (*p* < 0.001).

391 TCGA CRC samples showed various methylation levels (*β* value) of *SDC2* or *TFPI2* probes (Figure 1B). The methylation level of 391 CRC tissues was significantly higher than that of 45 normal tissues, either for the *SDC2* gene (0.479 ± 0.178 vs. 0.067 ± 0.018) or for the *TFPI2* gene (0.558 ± 0.149 vs. 0.161 ± 0.078).

A similar tendency was observed in the datasets GSE48684 and GSE79740 (Figures 1C,D), and the methylation level of both *SDC2* and *TFPI2* was higher in CRC than in normal tissue. However, no significant difference was detected between CRC and adenoma samples in GSE48684 (*p* = 0.79 for *SDC2* and *p* = 0.35 for *TFPI2*) (Figure 1C).

### MSP Could Efficiently Differentiate CRC, Adenoma, and Normal Samples

MSP assays were developed. The PCR reaction was considered effective if the Ct value of *ACTB* ≤ 36. The standard curve data showed that the amplification efficiency of the three genes (*SDC2*, *TFPI2*, and *ACTB*) was similar, ranging from 102 to 105% (Supplementary Figure S1). Furthermore, methylated primers and probes could only specifically amplify the positive templates (positive plasmids and the CRC cell line HT-29), but not the negative templates (negative plasmids and the control cell line Hacat) (Supplementary Figure S2).

**FIGURE 2:**
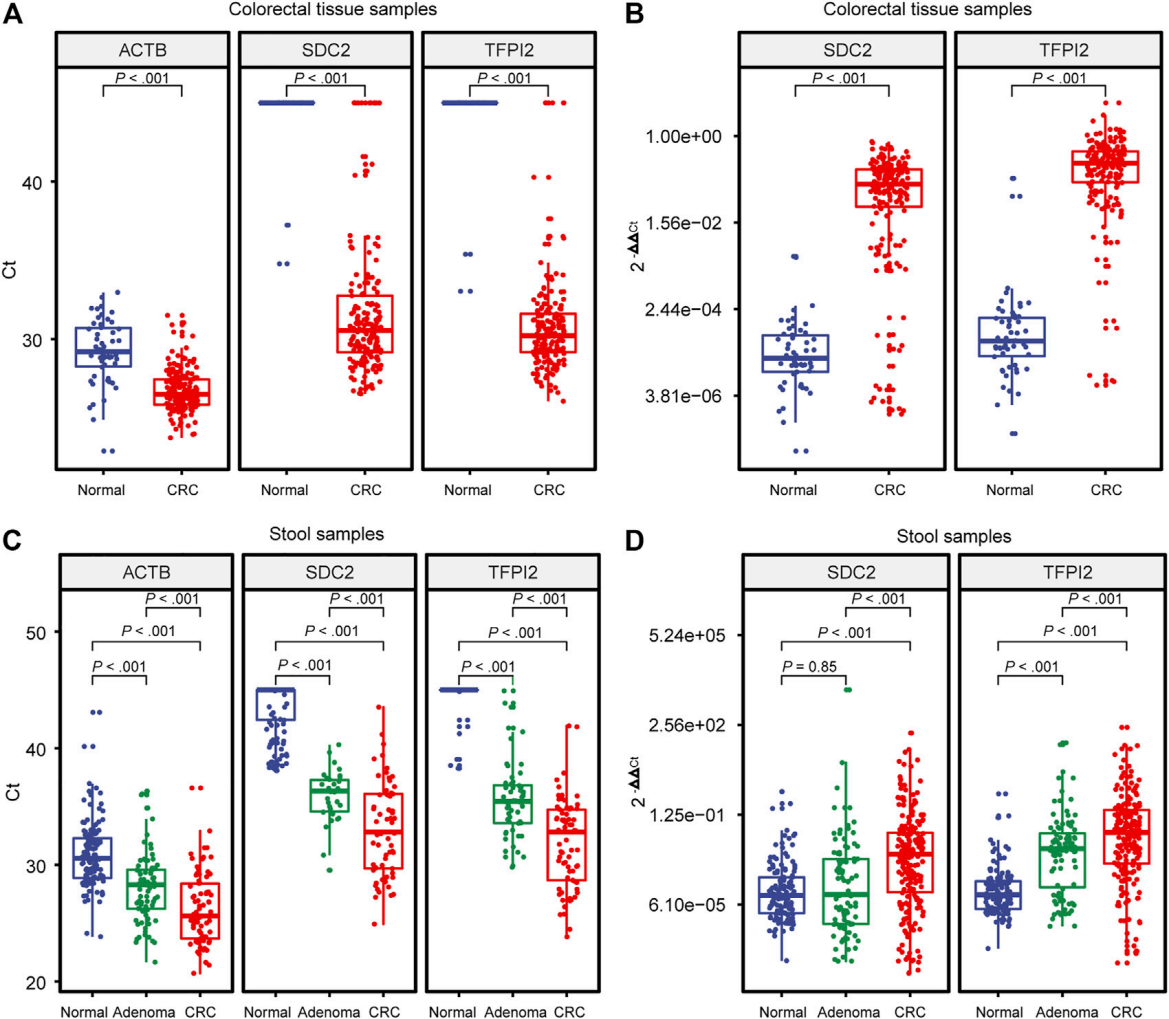
Distribution of Ct and 2^−ΔΔCt^ values generated by MSP. **(A)** and **(B)** are built on colorectal tissue specimens. **(C)** and **(D)** are built on stool specimens. Each dot indicates an individual specimen. The extremes of the boxes define the upper and lower quartiles, and the center lines define the median. Whiskers indicate 1.5×interquartile range (IQR). Beyond IQR are defined the outliers. Statistically significant differences were determined using the Mann–Whitney U test, with a significant level of *p* < 0.05 and a highly significant level of *p* < 0.01.

MSP assays were then evaluated with 238 tissue (184 CRC and 54 healthy control samples) and 696 stool (289 CRC, 190 adenoma, and 217 healthy control samples) specimens. Figure 2 shows the distribution of Ct values (Figures 2A,C) and $2^{-\Delta \Delta Ct}$ (Figures 2B,D) for CRC, adenoma, and normal control samples.

The endogenous reference gene *ACTB* showed a significant difference (*p* < 0.001) in Ct value, showing a tendency of normal > adenoma > CRC (Figures 2A,C). Since the Ct value of the endogenous reference *ACTB* is a function of the copy number of template DNA, in stool samples, it reflects the number of human exfoliated cells in the sample, and the result indicated that the number of human exfoliated cells in stool samples showed a tendency of CRC > adenoma > normal control.

Ct values of the tissue samples (Figure 2A) showed a highly significant difference (*p* < 0.001) between CRC and the control for either *SDC2* or *TFPI2*. Ct values of the stool samples showed highly significant differences in *SDC2* and *TFPI2* between CRC, adenoma, and control (Figure 2C), with a similar tendency of normal > adenoma > CRC.

Among stool samples, 55.3% CRC samples and 25.3% adenoma samples with an *SDC2* Ct value larger than 38 (false-negative) showed positive results in *TFPI2* MSP assays (Table 3).

**TABLE 3:** Methylation-positive rate (Ct ≤ 38) of TFPI2 within SDC2 methylation–negative (Ct>38) stool specimens.

Sample type	Tumor location	Frequency of specimens			TFPI2 methylation–positive rate (Ct ≤ 38)
		Positive	Negative	Total	
Carcinoma	Left colon	5	3	8	62.50%
	Right colon	1	3	4	25.00%
	Sigmoid colon	8	5	13	61.50%
	Rectal	7	6	13	53.80%
	Total	21	17	38	55.30%
Adenoma	Left colon	5	12	17	29.40%
	Right colon	1	16	17	5.90%
	Sigmoid colon	8	15	23	34.80%
	Rectal	7	19	26	26.90%
	Total	21	62	83	25.30%
Normal	Total	6	202	208	2.89%

The methylation level (ML) of a sample was estimated by the formula $ML=2^{-\Delta \Delta Ct}$ . In tissue sample testing, the $2^{-\Delta \Delta Ct}$ value showed a tendency of normal < cancer (*p* < 0.001) for either *SDC2* or *TFPI2* (Figure 2B). In stool samples, the methylation level of *TFPI2* was higher in CRC than in adenoma (*p* < 0.001) and in adenoma than in the normal group (*p* < 0.001) (Figure 2D, right). The methylation level of *SDC2* was significantly higher in cancer than in adenoma samples, but the difference in methylation level of *SDC2* between adenoma and normal samples was not significant (*p* = 0.85) (Figure 2D, left).

### Ct-Based Diagnosis Was Superior to ML-Based Diagnosis, and *SDC2*/*TFPI2*-Combined Detection Showed Better Diagnostic Performance Than *SDC2* Detection Alone

**FIGURE 3:**
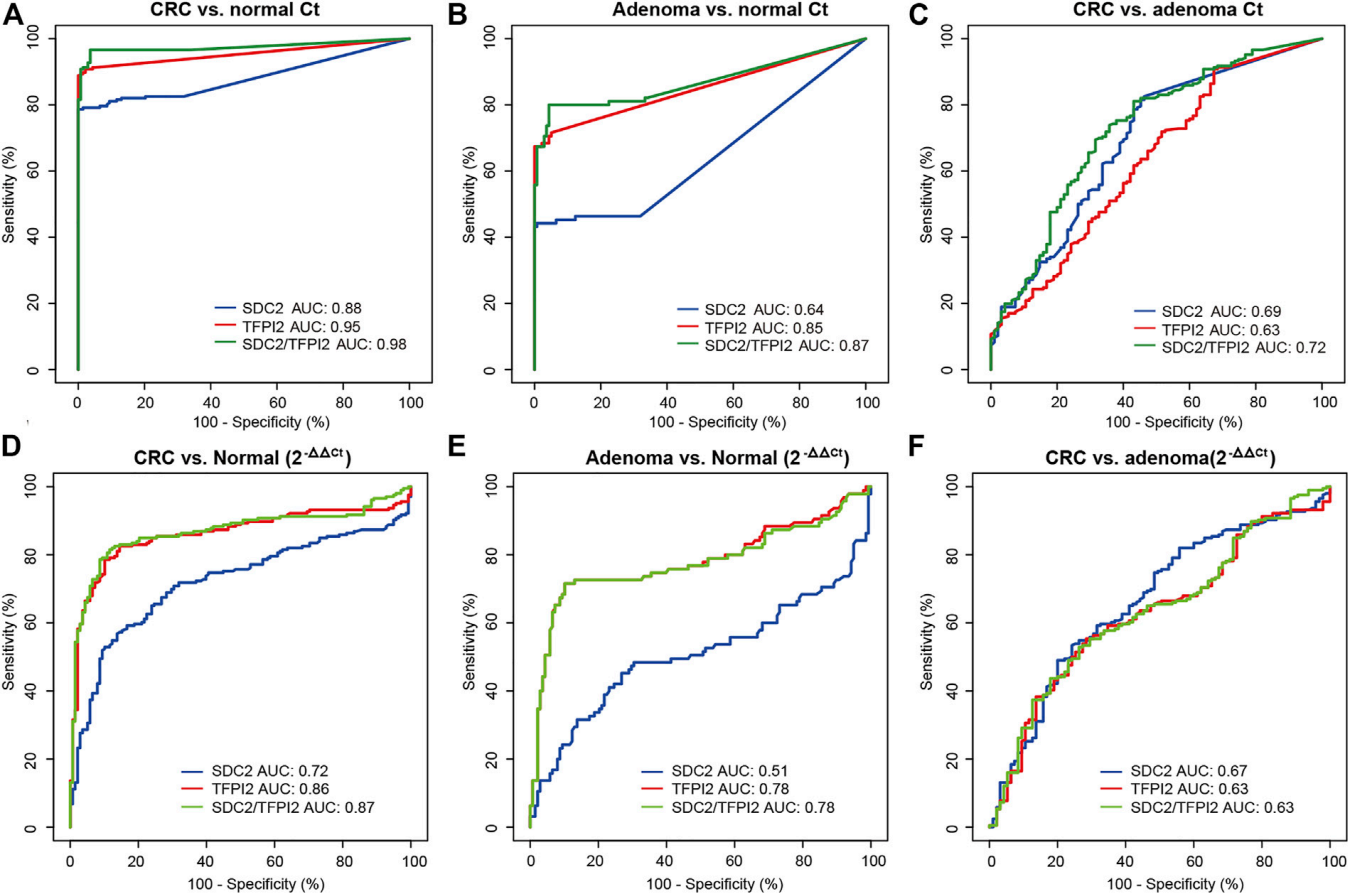
Diagnostic performances of methylation-specific PCR targeting *SDC2*, *TFPI2*, and SDC2/*TFPI2* in stool specimens. **(A–C)** are ROC curves based on Ct values, for cancer vs. normal, adenoma vs. normal, and cancer vs. adenoma, respectively. **(D–F)** are ROC curves based on ML values, for cancer vs. normal, adenoma vs. normal, and cancer vs. adenoma, respectively. Ct values are obtained in stool specimens by methylation-specific PCR. ML = 2-^ΔΔCt^, in which ΔΔCt = (Ct_*target*_ - Ct_*ACTB*_)_sample_ - (Ct_*target*_ - Ct_*ACTB*_)_positive control_. AUC means the area under the curve.

The diagnostic performance of *SDC2*, *TFPI2*, and *SDC2*/*TFPI2* in stool samples was evaluated by ROC curve analysis with colonoscopy as the gold standard and MSP result as the evaluation index (Figure 3 and Table 4). Figures 3A–C display the ROC curves based on Ct values and Figures 3D–F based on ML values.

**TABLE 4:** Diagnostic performance of methylation-specific PCR in stool specimens with the Ct value and ML as indicators^a^.

Marker	Indicator	Specificity (%)	Sensitivity (%)	AUC	AUC 95% CI	Group
*SDC2*	Ct	99.30	44.20	0.64	0.57 to 0.71	Adenoma vs. normal
*TFPI2*	Ct	100.00	67.40	0.85	0.81 to 0.89	
*SDC2/TFPI2*	Ct	95.70	80.00	0.87	0.81 to 0.92	
*SDC2*	Ct	100.00	78.60	0.88	0.85 to 0.92	Cancer vs. normal
*TFPI2*	Ct	100.00	88.80	0.95	0.93 to 0.97	
*SDC2/TFPI2*	Ct	96.40	96.60	0.98	0.96 to 0.99	
*SDC2*	Ct	54.70	81.60	0.69	0.63 to 0.75	Cancer vs. adenoma
*TFPI2*	Ct	32.60	90.80	0.63	0.59 to 0.70	
*SDC2/TFPI2*	Ct	64.20	73.80	0.72	0.67 to 0.78	
*SDC2*	ML	73.20	45.30	0.51	0.42 to 0.59	Adenoma vs. normal
*TFPI2*	ML	89.90	71.60	0.78	0.70 to 0.84	
*SDC2/TFPI2*	ML	89.90	71.60	0.78	0.72 to 0.85	
*SDC2*	ML	86.20	56.80	0.72	0.66 to 0.77	Cancer vs. normal
*TFPI2*	ML	89.90	78.60	0.86	0.82 to 0.90	
*SDC2/TFPI2*	ML	88.40	82.00	0.87	0.84 to 0.90	
*SDC2*	ML	75.80	53.40	0.67	0.61 to 0.72	Cancer vs. adenoma
*TFPI2*	ML	71.60	55.30	0.63	0.56 to 0.69	
*SDC2/TFPI2*	ML	73.70	52.90	0.67	0.62 to 0.74	

a ML = 2^−ΔΔCt^, in which ΔΔCt = (Ct_*target*_ - Ct_*ACTB*_)_sample_ - (Ct_*target*_ - Ct_*ACTB*_)_positive control_.

The Ct-based ROC curve analysis (Figure 3A; Table 4) showed that, for CRC vs. normal, the AUC value of *SDC2*/*TFPI2*-combined detection was 0.98 (95% CI: 0.96–0.99) with the specificity of 96.40% and sensitivity of 96.60%, while the AUC value of *SDC2* detection was 0.88 (95% CI: 0.85–0.92) with the specificity of 100% and sensitivity of 78.60%. For adenoma vs. normal (Figure 3B; Table 4), the AUC value of *SDC2*/*TFPI2*-combined detection was 0.87 (95% CI: 0.81–0.92) with the specificity of 95.70% and sensitivity of 80.00%, while the AUC value of *SDC2* detection was 0.64 (95% CI: 0.57–0.71) with the specificity of 99.30% and sensitivity of 44.20%. *SDC2*/*TFPI2*-combined detection showed better diagnostic performance than *SDC2* detection alone for CRC vs. normal and for adenoma vs. normal as well as for adenoma vs. CRC. The AUC value of *SDC2*/*TFPI2* for CRC vs. adenoma was 0.72, with the specificity of 64.20% and sensitivity of 73.80%, which was lower than that of CRC vs. normal or adenoma vs. normal.

When ML values were used as the detection index in ROC curve analysis, *SDC2*/*TFPI2*-combined detection also showed better diagnostic performance than *SDC2* detection in discrimination between cancer and normal, between adenoma and normal, and between adenoma and cancer. However, ML-based diagnostic performance was significantly lower than that of Ct-based diagnosis (Figure 3; Table 4).

### *TFPI2* Could Improve the Detection Sensitivity of *SDC2* Through Finding Cancer in Left Colon, Sigmoid Colon, and Rectum

**FIGURE 4:**
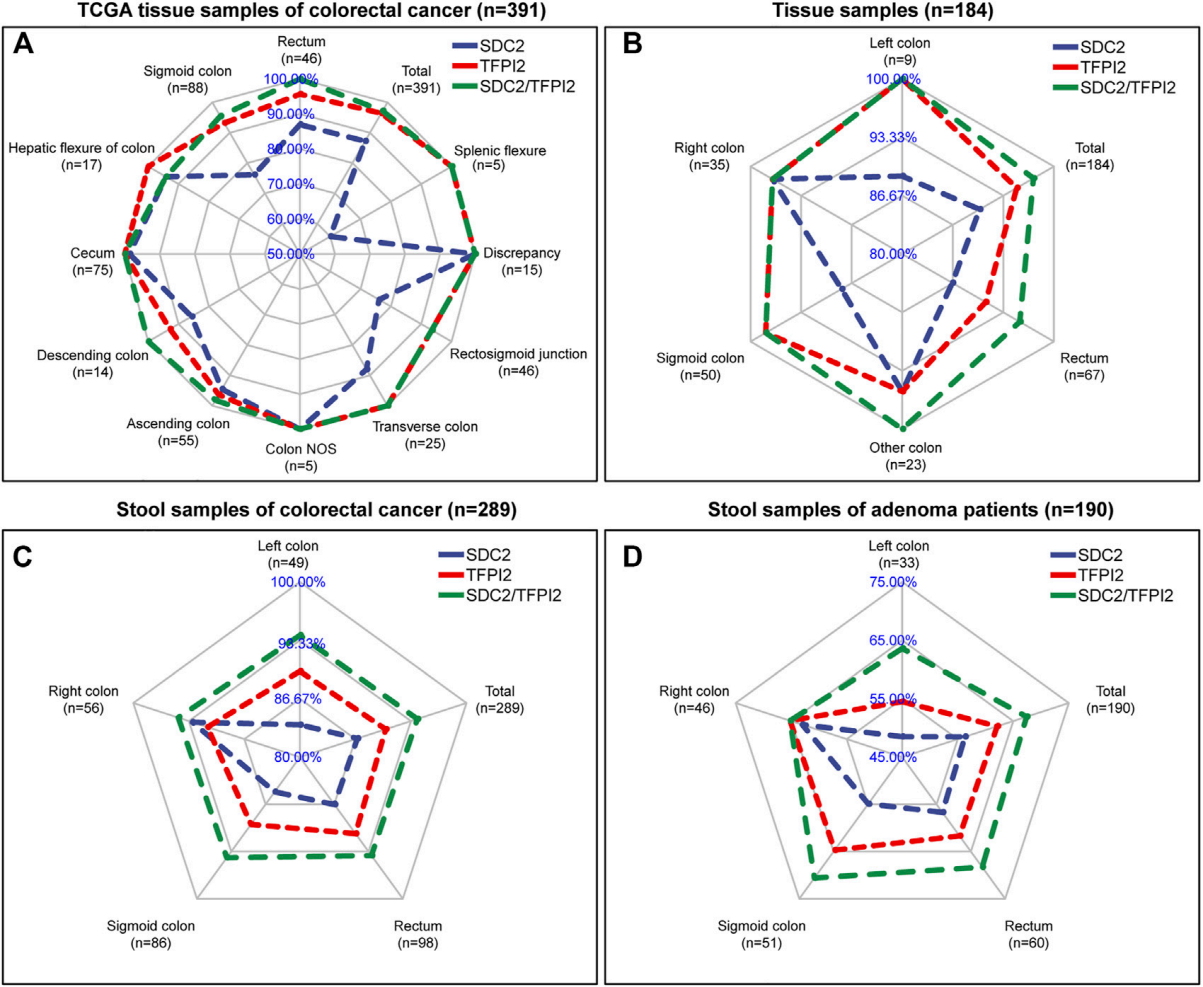
Comparison of the sensitivity of *SDC2*, *TFPI2*, and *SDC2*/*TFPI2* in detecting colorectal cancer of various locations. The blue line is related to the detection sensitivity of *SDC2*, the red line to that of *TFPI2*, and the green line to that of *SDC2*/*TFPI2* in combination. The cutoff value is β = 0.2 for **(A)** and Ct = 38 for **(B–D)**. **(A)** shows detection sensitivity based on β values generated by a 450k methylation chip in TCGA tissue specimens (n = 391 cancer and 45 normal samples). **(B)** shows detection sensitivity based on Ct values generated by methylation-specific PCR of the tissue specimens (n = 184) collected in this study. **(C)** shows detection sensitivity based on Ct values generated by methylation-specific PCR of stool specimens (n = 289) of patients with colorectal cancer collected in this study. **(D)** shows detection sensitivity based on Ct values generated by methylation-specific PCR of stool specimens (n = 190) of patients with colorectal adenoma collected in this study.

Figure 4 shows the sensitivity comparison of different detection methods (targeting *SDC2*, *TFPI2* alone and *SDC2*/*TFPI2* jointly) in different CRC sites. Figure 4A is based on 391 CRC specimens in TCGA. The sensitivity of *SDC2*/*TFPI2*-combined detection was 97.2%, while that of *SDC2* alone was 87.2%; the sensitivity difference between *SDC2* single gene detection (abbreviated to SD) and *SDC2*/*TFPI2* double gene detection (abbreviated to DD) was highly significant (*p* ≤ 0.01). The sensitivity difference between DD and SD varied with different locations. A highly significant improvement was found in the rectum (n = 46, DD/SD = 100.0%/87.0%, *p* ≤ 0.01), sigmoid colon (n = 88, DD/SD = 95.5%/76.1%, *p* ≤ 0.01), and rectosigmoid junction (n = 46, DD/SD = 93.5%/76.1%, *p* ≤ 0.01). Detection sensitivity was also increased for transverse colon (n = 25, DD/SD = 100%/88.0%), descending colon (n = 14, DD/SD = 100%/85.7%), and ascending colon (n = 55, DD/SD = 98.2%/94.5%) cancers though they were not statistically significant.

In 184 CRC tissue samples (Figure 4B), the overall sensitivity of DD was 97.3% while 90.2% for SD, and the difference between DD and SD was highly significant (*p* ≤ 0.01). The rectum (n = 67, DD/SD = 95.5%/86.6%), sigmoid colon (n = 50, DD/SD = 98.0%/88.0%), and left colon (n = 9, DD/SD = 100.0%/88.9%) showed the most significant improvement.

In 289 CRC stool samples (Figure 4C), the overall sensitivity of DD was 94.1%, while that of SD was 86.9%, and the difference between DD and SD was highly significant (*p* ≤ 0.001). The left colon (n = 49, DD/SD = 93.9%/83.7%), sigmoid colon (n = 86, DD/SD = 94.2%/84.9%), and rectum (n = 98, DD/SD = 93.9%/86.7%) showed the most significant improvement.

In 190 colorectal adenomas stool samples (Figure 4D), the sensitivity was 67.4% for DD while 56.3% for SD, and the difference between DD and SD was also highly significant (*p* ≤ 0.001). The left colon (n = 33, DD/SD = 66.6%/48.5%), sigmoid colon (n = 51, DD/SD = 70.6%/54.9%), and rectum (n = 60, DD/SD = 68.3%/56.7%) showed the most obvious sensitivity improvement.

Based on the above four datasets (Figure 4), it was found that the sensitivity of *SDC2*/*TFPI2*-combined detection was significantly higher than that of *SDC2* single gene detection. *TFPI2* could enhance the detection sensitivity of *SDC2* especially for cancer in the left colon, rectum, and sigmoid colon. Tissue samples and stool samples showed the same trend. *SDC2*/*TFPI2*-combined detection showed higher sensitivity not only to cancer samples but also to adenoma samples.

## Discussion

### Subsite Difference in Methylation Between *SDC2* and *TFPI2* and Its Application in Detection

CRCs that arise proximally or distally to the splenic flexure show differences in epidemiologic incidence, morphology, and molecular alterations (Huyghe et al., 2021). A previous investigation suggested that the degree of *SDC2* methylation in the left colon and the right colon may be different (McInnes et al., 2017). The difference in methylation of *TFPI2* and *SDC2* in different colorectal parts found in this study may be related to the etiologic heterogeneity of CRC.

*SDC2* is a member of the syndecan family and has been reported to play a critical role either as a tumor suppressor, such as in osteosarcoma (Mansouri et al., 2015), or as an oncogene, such as in breast cancer (Loftus et al., 2021). Hypermethylation of *SDC2* promoter region is a frequent epigenetic change in the development of colorectal neoplasms, and it has been successfully detected in several types of clinical specimens which include tissue, stool, and serum samples (Barták et al., 2017; Chen et al., 2019; Oh et al., 2013), making it an optimal target for developing a novel diagnostic kit for CRC early detection. In a previous study, the detection rate of *SDC2* methylation was 81.1 and 58.2% for CRC and adenoma, respectively, with the specificity of 93.3% (Niu et al., 2017), which was in agreement with our study (Figure 3; Table 4) and other research studies (Wang et al., 2020; Zhao et al., 2020), indicating that the sensitivity of *SDC2* for CRC and adenoma had room to be improved. To reduce the missed rate of detection, an additional marker which was complementary to *SDC2* might be an alternative way for this purpose.

*TFPI2* (tissue factor pathway inhibitor-2) is a Kunitz-type serine proteinase inhibitor that protects the extracellular matrix of cancer cells from degradation and inhibits *in vitro* colony formation and proliferation (Gerecke et al., 2015). *TFPI2* is frequently silenced in human hepatocellular carcinoma via epigenetic alterations, including promoter methylation and histone deacetylation (Wong et al., 2007). Glockner et al. demonstrated that the methylation of *TFPI2* was a frequent event in human colorectal cancer using a gene expression array–based strategy (Glöckner et al., 2009).

We firstly demonstrated that there was a subsite difference in colorectal methylation between *TFPI2* and *SDC2*, and as high as 88.0% *SDC2* hypomethylated CRC samples retrieved from TCGA were *TFPI2* hypermethylated (Table 2), in agreement with the result from the MSP assays in stool samples (Table 3). The results of this study indicated that *TFPI2* could improve the detection sensitivity of *SDC2* through finding CRC in the left colon, sigmoid colon, and rectum (Figure 4). Sigmoid colon cancer and rectal cancer have a high incidence worldwide (Meza et al., 2010; Wang et al., 2021), and the sensitivity improvement would be of great benefit to the overall CRC detection.

### Diagnostic Performance of *SDC2*/*TFPI2* and Advantage Over the Present Techniques

It is very important to develop a stool DNA methylation test that is sensitive to detect early-stage CRC and precancerous lesions for effective surgical and therapeutic interventions. In the current multicenter clinical study, *SDC2*/*TFPI2*-joined detection demonstrated an overall sensitivity for all CRCs at 96.6% with the specificity at 96.4%, and the sensitivity for adenoma was as high as 80%, in contrast to the sensitivity of 30% for adenoma by the fecal occult blood test with high-sensitivity guaiac (gFOBT), or the fecal immunochemical test (FIT) (Graser et al., 2009), which are non-invasive detection methods that are most commonly used in clinical practice at present. In our study, the methylation level of CRC and adenoma was statistically different in stool samples (Figures 2C,D); however, *SDC2*/*TFPI2* did not differentiate CRC and adenoma well enough (Figure 3 and Table 4), which meant that the methylation pattern of adenoma was more similar to that of CRC. According to ACG guidelines (Shaukat et al., 2021), CRC screening efforts are directed toward removal of adenomas and sessile serrated lesions and detection of early-stage CRC; therefore, as long as the markers can effectively distinguish between normal and adenoma samples, it is beneficial to classify precancerous adenoma and cancer samples together as “positive samples,” so that if a sample is methylation positive, further colonoscopy and pathological examination can be performed. Once the adenoma is effectively treated, the chance of developing into cancer will be greatly reduced, which is beneficial to reducing the incidence of cancer.

High performance of *SDC2*/*TFPI2*-joined detection in this study derived from a series of technical improvement, including stool DNA preservation against DNA degradation in stool, sequence-specific capture technology based on magnetic beads which effectively eliminated background noise from massive amounts of contaminating plant, animal, and bacterial genomic DNA in MSP assays, and optimized primers and probe sets as well as assay conditions which are also potential contributors to the varied sensitivity and specificity. As a non-invasive detection method, *SDC2*/*TFPI2*-joined detection in stool samples is safe and can be operated easily, avoiding bowel preparation and possible cross-infection during colonoscopy.

### Ct Value–Based Method Is Superior to ML-Based Assay

The results showed that the Ct value instead of 2^−ΔΔCt^ as the detection index could improve the detection accuracy of adenoma and CRC (Figure 3). We found that the CRC, adenoma, and control were different in the stool samples not only in the methylation level as measured by 2^−ΔΔCt^ but also in the number of human exfoliated cells reflected by the Ct value of *ACTB* (Figure 2). The Ct value of *ACTB* in cancer samples was smaller than that in adenoma samples and further smaller than that in normal control samples (*p* < 0.001), indicating that the number of exfoliated cells in CRC stool samples was significantly more than that in adenoma samples and further more than that in normal control samples. The difference of methylation level and in addition the number of exfoliated cells resulted in the better sensitivity of Ct than 2^−ΔΔCt^ as the detection index. This was in agreement with other studies which also used the Ct value as the diagnostic index (Kim et al., 2021; Zhao et al., 2021).

### Highlights and Shortcomings

Despite that many methylation-based methods for CRC diagnosis have been reported, there exist some highlights in our study. Firstly, we identified *TFPI2* through whole-genome screening, significantly outperforming the well-established biomarker *SDC2* in CRC detection. Secondly, five populations from Asian and Euro-American regions and two specimen types (tissue and stool) were involved (Table 1), totally including 1,034 CRC patients, 232 adenoma patients, and 367 normal individuals, covering different colorectal sites and stages. Thirdly, three indexes (*β* value, Ct value, and 2^−ΔΔCt^ value) were evaluated and compared (Tables 2–4). The Ct value was a suitable indicator, being simple to operate and having better performance than the 2^−ΔΔCt^ value.

However, there are still certain limitations associated with our current investigation. First, a larger scale validation is required to accurately assess the effectiveness. The number of cases of advanced adenomas, in particular the pathology information regarding villous and serrated adenomas, is limited, hence lacking sufficient power to accurately estimate the test’s sensitivity and to perform further covariate analysis of these precancerous lesions.

### Conclusions

*TFPI2* can improve the sensitivity of *SDC2* methylation–specific detection of colorectal tumorous lesions while maintaining high specificity, in particular reducing the missed detection of left colon cancer and adenoma. As a non-invasive detection method, the dual detection of *SDC2*/*TFPI2* will be an easy and precise screening tool for colorectal cancer and its precancerous lesions.

## Data Availability

The raw data supporting the conclusions of this article will be made available by the authors, without undue reservation.

## Supplementary Material

Supplementary Figure S1 Amplification plots and standard curves. (A,C and E) are the amplification plots for ACTB, SDC2, and TFPI2 in two duplicates, respectively. (B, D and F) are the amplification standard curves for ACTB, SDC2, and TFPI2, respectively. Eff% indicates the PCR amplification efficiency, Y-inter represents Y-interception, and R2 means the determinant coefficient.

Supplementary Figure S2 PCR amplification curves of the positive and negative standards for the target genes (SDC2 and TFPI2) and the endogenous reference gene ACTB. (A) was from the positive (red lines) and negative (black lines) controls of plasmids, respectively. (B) was from cell lines of HT-29 and Hacat.

## Abbreviations

CRC, colorectal cancer; FOBT, fecal occult blood test; FIT, fecal immunochemical test; MSP, methylation-specific PCR; TCGA, The Cancer Genome Atlas; ROC, receiver-operating characteristic; AUC, area under the curve; GEO, Gene Expression Omnibus; 95% CI, 95% confidence interval; ML, methylation level which is defined as ML = 2^−ΔΔCt^; SD, single detection of *SDC2*; DD, double detection of *SDC2* and *TFPI2*.
