# Supplementary figures and images for: Methylation of SDC2/TFPI2 and Its Diagnostic Value in Colorectal Tumorous Lesions
Source: Front Mol Biosci. 2021 Dec 22;8:706754. doi: 10.3389/fmolb.2021.706754 (PMC8729808; doi:10.3389/fmolb.2021.706754)

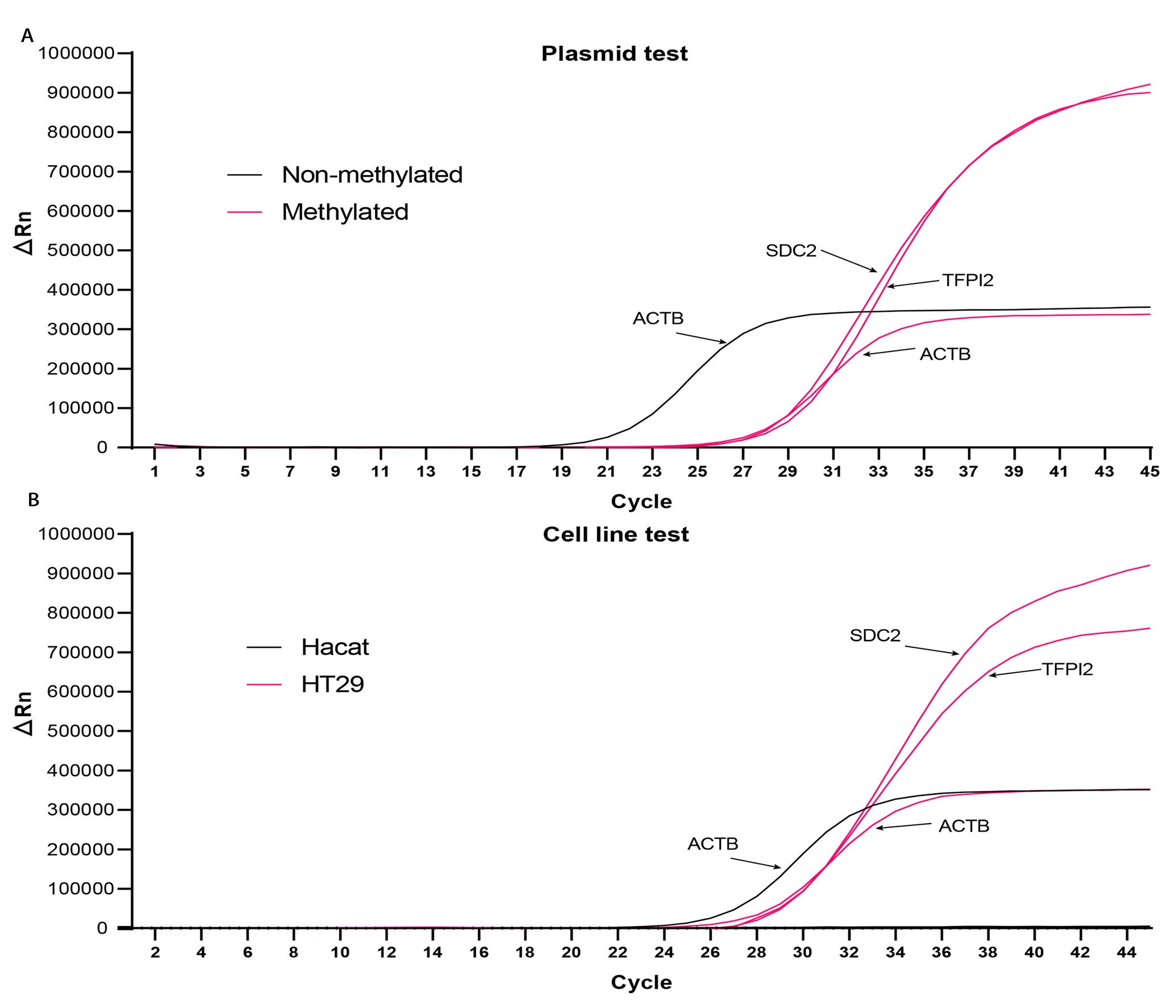

Supplement: Supplementary file 3 [file Image2.TIF]

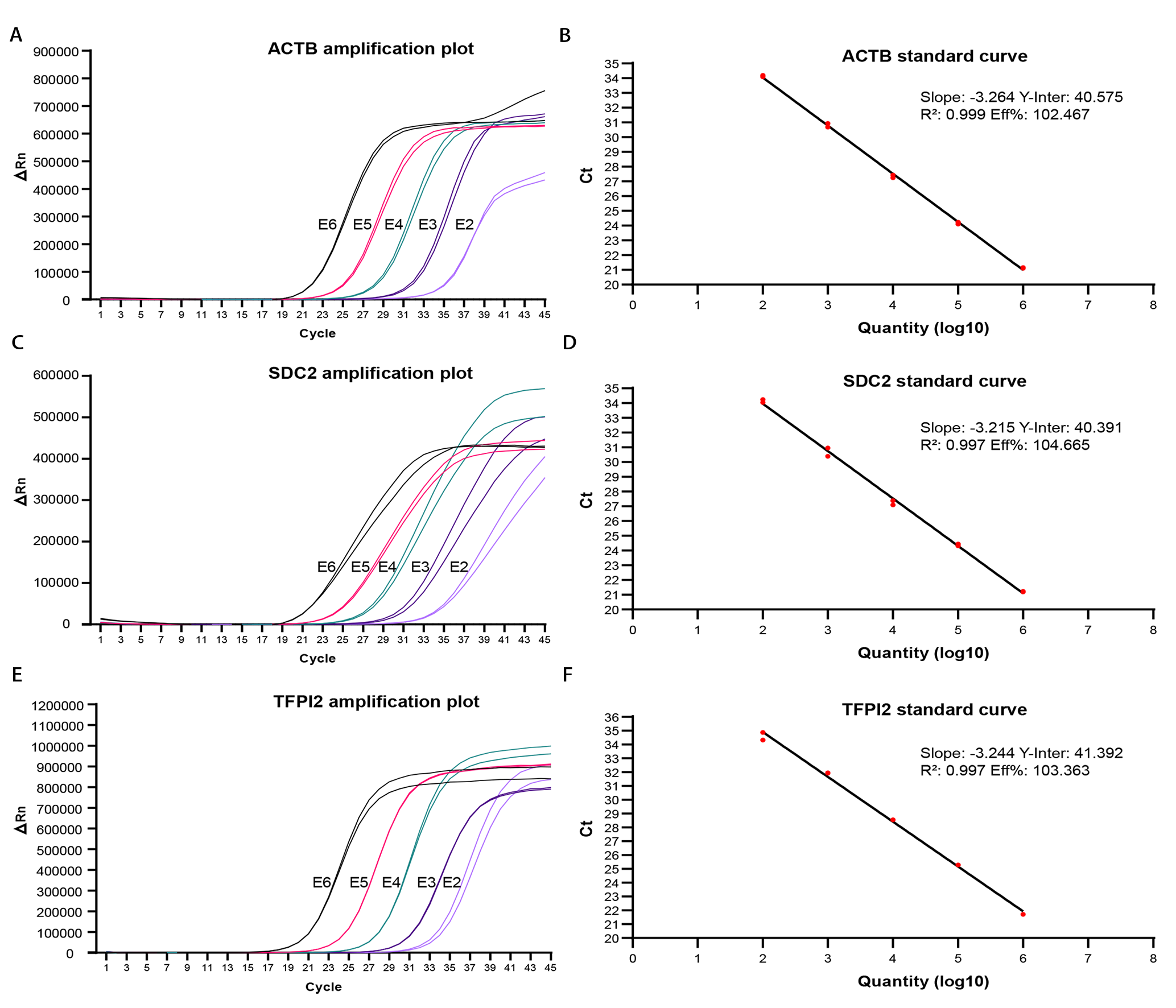

Supplement: Supplementary file 4 [file Image1.TIF]
